# Insulin Signaling in the Peripheral and Central Nervous System Regulates Female Sexual Receptivity during Starvation in *Drosophila*

**DOI:** 10.3389/fphys.2017.00685

**Published:** 2017-09-08

**Authors:** Sébastien Lebreton, Mikael A. Carlsson, Peter Witzgall

**Affiliations:** ^1^Division of Chemical Ecology, Department of Plant Protection Biology, Swedish University of Agricultural Sciences Alnarp, Sweden; ^2^Department of Zoology, Stockholm University Stockholm, Sweden

**Keywords:** mating behavior, feeding state, insulin, fruitless, mushroom bodies

## Abstract

Many animals adjust their reproductive behavior according to nutritional state and food availability. *Drosophila* females for instance decrease their sexual receptivity following starvation. Insulin signaling, which regulates many aspects of insect physiology and behavior, also affects reproduction in females. We show that insulin signaling is involved in the starvation-induced reduction in female receptivity. More specifically, females mutant for the insulin-like peptide 5 (*dilp5*) were less affected by starvation compared to the other *dilp* mutants and wild-type flies. Knocking-down the insulin receptor, either in all fruitless-positive neurons or a subset of these neurons dedicated to the perception of a male aphrodisiac pheromone, decreased the effect of starvation on female receptivity. Disrupting insulin signaling in some parts of the brain, including the mushroom bodies even abolished the effect of starvation. In addition, we identified fruitless-positive neurons in the dorso-lateral protocerebrum and in the mushroom bodies co-expressing the insulin receptor. Together, our results suggest that the interaction of insulin peptides determines the tuning of female sexual behavior, either by acting on pheromone perception or directly in the central nervous system.

## Introduction

Adjusting reproductive behavior to nutrient availability is a common feature in many animals. Females of many animal species decrease their sexual receptivity to male courtship in response to food deprivation (Kauffman and Rissman, 2004; Pierce et al., 2007; Franssen et al., 2008; Lebreton et al., 2015). In the fruit fly *Drosophila melanogaster*, feeding, and mating are strongly interconnected. Mating modifies food preference in females (Carvalho et al., 2006; Walker et al., 2015) while feeding regulates their mating behavior (Lebreton et al., 2015, 2016). The molecular mechanisms regulating feeding behavior after mating are fairly well described and involve the transfer of a male component during copulation called Sex Peptide (Carvalho et al., 2006; Ribeiro and Dickson, 2010; Walker et al., 2015). On the other hand, the mechanisms by which feeding and starvation regulate sexual receptivity remain unknown.

One potential system that mechanistically links these two behaviors is insulin signaling as it not only regulates feeding (Broughton et al., 2005; Slaidina et al., 2009; Lebreton et al., 2014) but also various aspects of reproduction such as female attractiveness, egg-laying and remating rate (Yang et al., 2008; Wigby et al., 2011; Kuo et al., 2012). Although, insulin signaling is not necessary for virgin females to be receptive when fed (Wigby et al., 2011; Sakai et al., 2014; Lebreton et al., 2015; Watanabe and Sakai, 2015), so far no study tested the role of this signaling pathway on the sexual behavior of females undergoing a period of starvation. Considering the conserved effect of starvation on sexual receptivity on the one hand, and the fact that insulin signaling both responds to nutrition and regulates reproductive behavior on the other hand, we expected insulin signaling to modulate female sexual receptivity during starvation.

Eight insulin-like peptides (DILP1-8) have been characterized in *D. melanogaster* while only one insulin receptor is known (InR; Nässel et al., 2013, 2015). DILP2, 3, and 5 are produced in specific cells in the pars intercerebralis called insulin-producing cells (IPCs). In addition, DILP5 is expressed in follicle cells of ovaries and principal cells in renal tubules (Ikeya et al., 2002). DILP6 is, on the other hand, produced by adipose cells (Slaidina et al., 2009). In contrast to other DILPs, DILP7, and DILP8 have been suggested to be more related to relaxin peptides than insulin peptides (Yang et al., 2008; Grönke et al., 2010; Garelli et al., 2015). Whereas, it is unknown whether DILP7 acts through the same InR, it has recently been shown that DILP8 acts via a specific relaxin receptor (Garelli et al., 2015). In addition, two DILPs are expressed almost exclusively during larval stages: DILP1 and DILP4 (Nässel et al., 2015). These different DILPs seem to interact to regulate the fly behavior and metabolism (Grönke et al., 2010; Kannan and Fridell, 2013; Nässel et al., 2013).

DILPs regulate the activity of neuronal circuitries to match behaviors with nutritional status (Wu et al., 2005a,b). Several neuronal networks have been shown to be involved in female sexual receptivity. For instance, neurons expressing the *fruitless* (*fru*) gene are necessary for females to be sexually receptive (Demir and Dickson, 2005; Kvitsiani and Dickson, 2006). Similarly, a subset of neurons expressing *doublesex* (*dsx*) regulates receptivity, independently of *fru* (Zhou et al., 2014). Both *fru* and *dsx* encode for a transcription factor that are spliced differently in males and females and account for sexually dimorphic traits (Siwicki and Kravitz, 2009). *fru* is expressed in some pheromone-sensing olfactory sensory neurons (OSNs) expressing the odorant receptors Or67d and Or47b (Stockinger et al., 2005). Or67d detects the male aphrodisiac pheromone *cis*-vaccenyl acetate (cVA) while Or47b responds to methyl laurate, a compound produced by flies of both sexes (Dweck et al., 2015). Both OSNs expressing Or67d and Or47b have been shown to modulate female receptivity (Kurtovic et al., 2007; Sakurai et al., 2013). In addition to these neurons, an early study found a group of cells in the dorsal anterior brain to be necessary and sufficient to induce receptivity (Tompkins and Hall, 1983), though these cells have never been precisely characterized. Interestingly, these cells seem to be different from those required to perform courtship in males (Tompkins and Hall, 1983), suggesting that they are probably not *fru*-positive. In young virgin females, acquisition of sexual receptivity is paralleled by the growth of the ovaries and the corpora allata, endocrine glands producing the juvenile hormone (JH, Manning, 1967). Both JH and the corpora allata have been shown to be involved in this switch in female receptivity (Manning, 1967; Ringo et al., 1991). Interestingly, the corpora allata activity is modulated by the insulin pathway (Tu et al., 2005; Belgacem and Martin, 2007). However, whether or not insulin acts on these structures to regulate the female sexual receptivity is unknown.

In conclusion, despite the fact that insulin is known to both regulate reproductive behavior and respond to food intake/deprivation, its effect in the starvation-induced reduction in female receptivity has not yet been established. We hypothesize that, during starvation, insulin signaling acts on specific neuronal networks to reduce sexual receptivity in order to match nutrition and reproduction. In the present study, we first carefully analyzed the effect of starvation in female mating behavior in *Drosophila*. We then tested the role of insulin by analyzing the behavior of females deficient for seven different DILPs. Finally, in an attempt to identify some structures on which insulin signaling acts to regulate sexual behavior, we knocked down InR in various brain parts known to be involved in mating behavior or behavioral modulation.

## Materials and methods

### Insects

*Drosophila* flies were reared on a sugar-yeast-cornmeal medium diet under a 12:12 h L:D photoperiod. Virgin flies were collected within 6 h following adult emergence. They were anesthetized with CO_2_ and separated by sex under a microscope. Flies of the same sex (males and females) were then kept in 30-ml plastic tubes with fresh diet for 3 days before behavioral experiments. For starvation, females were transferred to a humidified piece of cotton for 1, 2, or 3 days before being tested. Flies starved for 3 days were allowed to feed up to 6 h following adult emergence, before they were collected for experiments.

For behavioral experiments, the Dalby strain was used as a wild-type strain (Ruebenbauer et al., 2008). In order to test the effect of the different insulin-like peptides on female receptivity, mutant females for single dilps (dilp1 to 7) or multiple dilps (dilp2-3,5) were used. DILP8, which has been shown to be more related to relaxin peptides than insulin and to act via its own receptor, was not included in this study. In order to identify structures involved in this behavioral modulation, we manipulated insulin signaling in specific parts of the body. For this purpose, InR was knocked-down by crossing a line expressing an InR RNAi (uas-InR RNAi) to lines expressing specific Gal4 drivers (Fru-Gal4 for Fruitless-positive neurons, Aug21-Gal4 for the corpora allata, OK107-Gal4 for the mushroom bodies and Or67d-Gal4 for Or67d-expressing sensory neurons). All mutant and transgenic lines used and their origins are listed in Table 1.

**Table 1:** Mutant and transgenic fly lines used in this study.

**Fly line name**	**Stock #**	**References**
dilp1	BDSC #30880	Grönke et al., 2010
dilp2	BDSC #30881	Grönke et al., 2010; Okamoto and Nishimura, 2015
dilp3	BDSC #30882	Grönke et al., 2010; Okamoto and Nishimura, 2015
dilp4	BDSC #30883	Grönke et al., 2010
dilp5	BDSC #30884	Grönke et al., 2010; Okamoto and Nishimura, 2015
dilp6	BDSC #30885	Grönke et al., 2010
dilp7	BDSC #30887	Grönke et al., 2010
dilp2-3,5	BDSC #30889	Grönke et al., 2010; Okamoto and Nishimura, 2015
uas-InR RNAi	VDRC #992	Tang et al., 2011; Lebreton et al., 2015; Okamoto and Nishimura, 2015
fru-Gal4	BDSC #30027	Hu et al., 2014; Peng et al., 2015
Aug21-Gal4	BDSC #30137	Ádám et al., 2003
OK107-Gal4	BDSC #854	Manoli et al., 2005; Tanaka et al., 2008; Bräcker et al., 2013
Or67d-Gal4	BDSC #23906	Lebreton et al., 2015

*BDSC, Bloomington Drosophila Stock Center; VDRC, Vienna Drosophila Resource Center*.

### Mating behavior

One random wild-type (Dalby) 3d-old fed male was introduced together with a virgin female (either wild-type, mutant or transgenic) under a small round inverted plastic cup (45 mm in diameter, 30 mm high) placed on a clean glass plate. Wild-type females were either fed or starved for 1, 2, or 3 days (*n* = 52, 54, 77, and 47, respectively). Mutant and transgenic females were tested fed or 2-day starved. For mutant and transgenic flies, 33–50 couples were tested for each genotype and feeding condition in order to obtain 30–35 replicates of successful courtship.

Flies were observed for 60 min. Male courtship, courtship latency (time between the beginning of the test and courtship initiation), mating latency (time between the beginning of courtship and copulation initiation) and mating duration were recorded. To estimate female sexual receptivity, only cases where courtship from males was observed were taken into account. The percentage of females accepting to mate with a courting male within the 60-min period was then calculated for each 5-min interval.

Percentages of courting males were analyzed using a χ^2^-test. Courtship latency, mating latency and mating duration were analyzed using a Generalized Linear Model (GLM) with a Gamma family.

For each wild-type, mutant and transgenic line, an estimated decrease of sexual receptivity was calculated. For this purpose, the area under curve (AUC) was used as a proxy for sexual receptivity (R: auc function in kulife package). The percentage of decrease between AUC of fed and starved females was then calculated.

Regarding the percentage of mating, repeated measurements on the same flies over time were treated as pseudo-replicates and therefore analyzed using a Linear Mixed-effect Model (GLMM, R: glmer function in lme4 package) with a binomial distribution and the “time” factor as a random effect (Crawley, 2007). Similarly, the percentage by which mating was decreased between fed and starved females was compared between mutant and wild-type flies using a GLMM with a Gamma family. When GLM and GLMM showed a significant effect of the treatment or genotype, the test was followed by a multiple comparison test with a FDR correction method (R: glht function in multcomp package).

Statistical analyses were performed in R (R 2.1.1, R Development Core Team, Free Software Foundation Boston, MA, USA).

### Immunostaining

Standard immunohistochemical methods were used as earlier described in detail (Carlsson et al., 2010). In brief, dissected brains were fixed for 4 h at 4°C in 4% paraformaldehyde (PFA) and subsequently washed several times in phosphate buffer. The brains were then preincubated overnight in incubation buffer containing 0.01 M phosphate-buffered saline (PBS), 0.25% BSA, 0.25% Triton-X and 3% normal goat serum. Then the brains were incubated with a cocktail of the primary antibodies, mouse monoclonal GFP antibody (1:1,000, Molecular Probes, Invitrogen) and rabbit anti InR (1:1,000, #3021, Cell Signaling Technology) for 72 h at 4°C under gentle agitation. For detection of primary antisera, Alexa goat anti-rabbit 488 and Alexa goat anti-mouse 546 (Invitrogen) were used at a dilution of 1:500 at 4°C overnight, washed in PBS-Tx and PBS and finally mounted in 80% glycerol in PBS.

### Imaging

Brains were imaged with a Zeiss LSM 780 confocal microscope (Zeiss Jena, Germany) and stacked images were processed using ZEN 2011 software (Zeiss) and edited for intensity and contrast in Adobe Photoshop CS6.

## Results

### Starvation regulates female sexual receptivity

**Figure 1:**
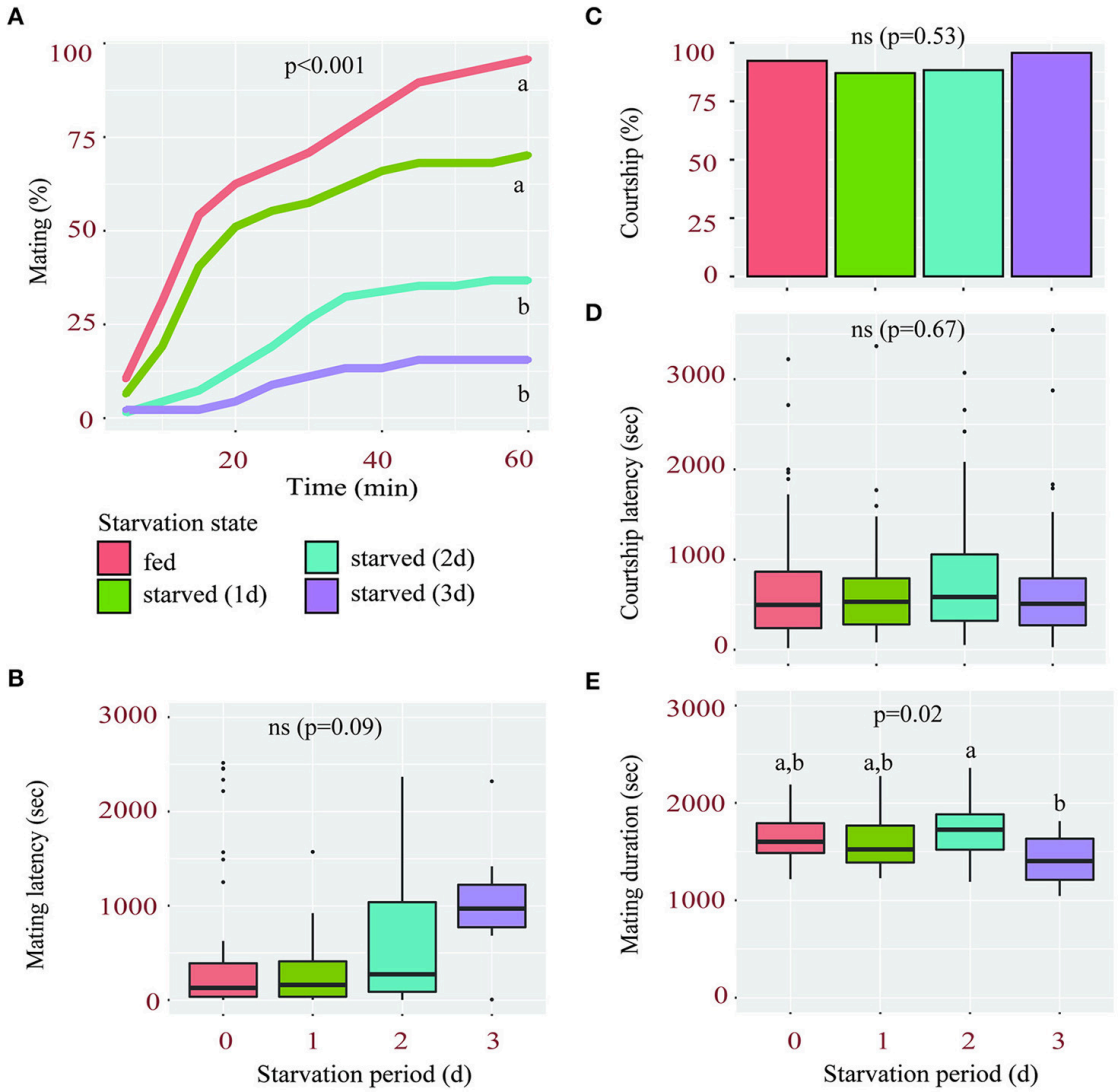
Starvation affects female sexual receptivity. **(A)** Percentage of wild-type females accepting to mate with a courting male during a 1 h-period according to their starvation state (fed or starved for 1, 2, or 3 days). Linear Mixed-Effect Model (*p* < 0.001), different letters show statistical significant differences between starvation states. **(B)** Mating latency (time from courtship initiation to mating) of fed and starved females. **(C–E)** Percentage of males courting fed and starved females **(C)**, their corresponding courtship latency **(D)** and mating duration **(E)**.

We found that female sexual receptivity was significantly reduced after a period of starvation. When fed, 96% of virgin females mated within 1 h of being paired with a wild type male. However, this percentage dropped to 70, 37, and 16% after a starvation period of 1, 2, and 3 days, respectively (Figure 1A). Although, starved females also tended to have longer mating latencies, this effect was not significant (Figure 1B).

The drop in sexual receptivity observed in starved females was likely not due to changes in male courtship or mating behavior. First, neither the percentage of courting males (Figure 1C) nor their courtship latency (Figure 1D) was significantly affected by the starvation state of the females they were exposed to, suggesting that all females elicited a similar intensity of courtship. Second, the mean mating duration, a male-regulated aspect of copulation reflecting his investment (Bretman et al., 2009; Wigby et al., 2009), was not significantly shorter in any of the starvation conditions compared to fed females (Figure 1E). Taken together, these findings suggest that the starvation-induced reduction in female receptivity is likely due to altered molecular processes within the female and not reduced male interest.

### The decrease of sexual receptivity after starvation is less pronounced in *dilp5* mutants than in other *dilp* mutants and wild-type flies

Given the preponderant role of insulin signaling in response to changes in nutritional states, we then tested whether it could be involved in the regulation of sexual receptivity during starvation. We therefore investigated the mating behavior of seven *dilp* mutant females.

**Figure 2:**
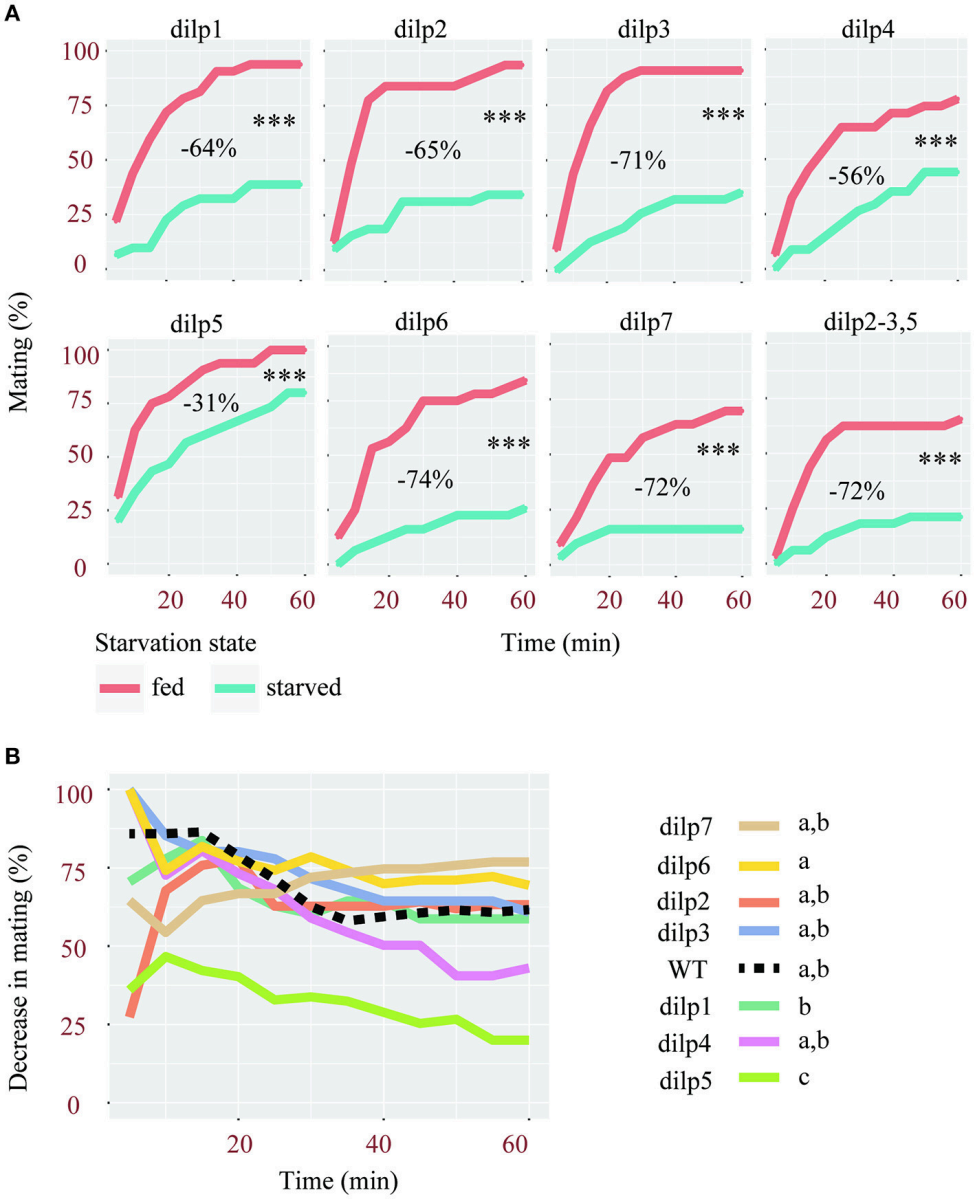
Mutations of single *dilps* differentially affect female receptivity. **(A)** Effect of starvation on the receptivity of females, mutant for different *dilps*. Tested females were either fed or starved for 2 days. ^***^p < 0.001 (Linear Mixed-Effect Model). For each mutant line is indicated the decrease of sexual receptivity after starvation, estimated based on the area under curves (AUC). **(B)** Comparison of the percentage of decrease in mating after starvation in single *dilp* mutant and wild-type females (Linear Mixed-Effect Model, *p* < 0.001), different letters show statistical significant differences between fly lines.

Similar to wild type flies, females carrying a mutation in either one or multiple *dilp* genes showed significantly reduced sexual receptivity after 2 days of starvation (Figure 2A). Although, the sexual receptivity of fed females varies between lines, the magnitude by which starvation affects sexual receptivity in these lines is overall similar. Indeed, the sexual receptivity of *dilp1, dilp2, dilp3, dilp6*, and *dilp7* mutants is decreased by 64 to 72%. This is similar to what was observed in wild-type flies (Figure 1A, 66%), even though their genetic background differs. Females mutant for *dilp4* showed a reduction of 56% of their sexual receptivity. *dilp5* mutant females were the least affected with a decrease of only 31%. Indeed, the percentage by which mating was decreased after starvation was significantly lower in dilp5 mutants compared to other single *dilp* mutants and wild-type flies (Figure 2B). Interestingly, the effect of the lack of DILP5 was not present in a *dilp2-3* mutant background (Figure 2, *dilp2-3,5* mutants: −72%).

In conclusion, among all flies deficient for one or several DILPs, only the lack of DILP5 significantly reduced the effect of starvation.

### Disrupting the insulin signaling in specific neuronal circuitries inhibits the effect of starvation on sexual receptivity

Although, the effect was less pronounced in *dilp5* mutants, the sexual receptivity of all *dilp* mutants was significantly reduced after 2 days of starvation, suggesting that insulin signaling is not involved. However, compensatory mechanisms exist among DILPs (Broughton et al., 2008; Grönke et al., 2010), which may have led to a masking effect when the expression of a single *dilp* was abolished. Since all DILPs act via a single insulin receptor (InR), disrupting InR and therefore all DILP signaling could reveal hidden effects. We therefore knocked-down InR in specific parts of the body, using specific Gal4 drivers. These drivers were chosen because they target specific sites known to regulate different aspects of *Drosophila* behavior. Fruitless-positive neurons (Fru-Gal4) are involved in sexually dimorphic behavior (Siwicki and Kravitz, 2009), Or67d-expressing OSNs (a sub-population of Fruitless-positive neurons, Or67d-Gal4) detect the male aphrodisiac pheromone cVA (Datta et al., 2008), cells of the corpora allata (Aug21-Gal4) are necessary for young virgin females to become sexually receptive (Manning, 1966, 1967) and the mushroom bodies (OK107-Gal4) are involved various behavioral processes such as courtship and decision making (Zars, 2000). Of note, OK107-Gal4 also drives expression to some extent in the pars intercerebralis, optic lobes, the subesophageal ganglion, the tritocerebrum, and the antennal lobes (Aso et al., 2009).

**Figure 3:**
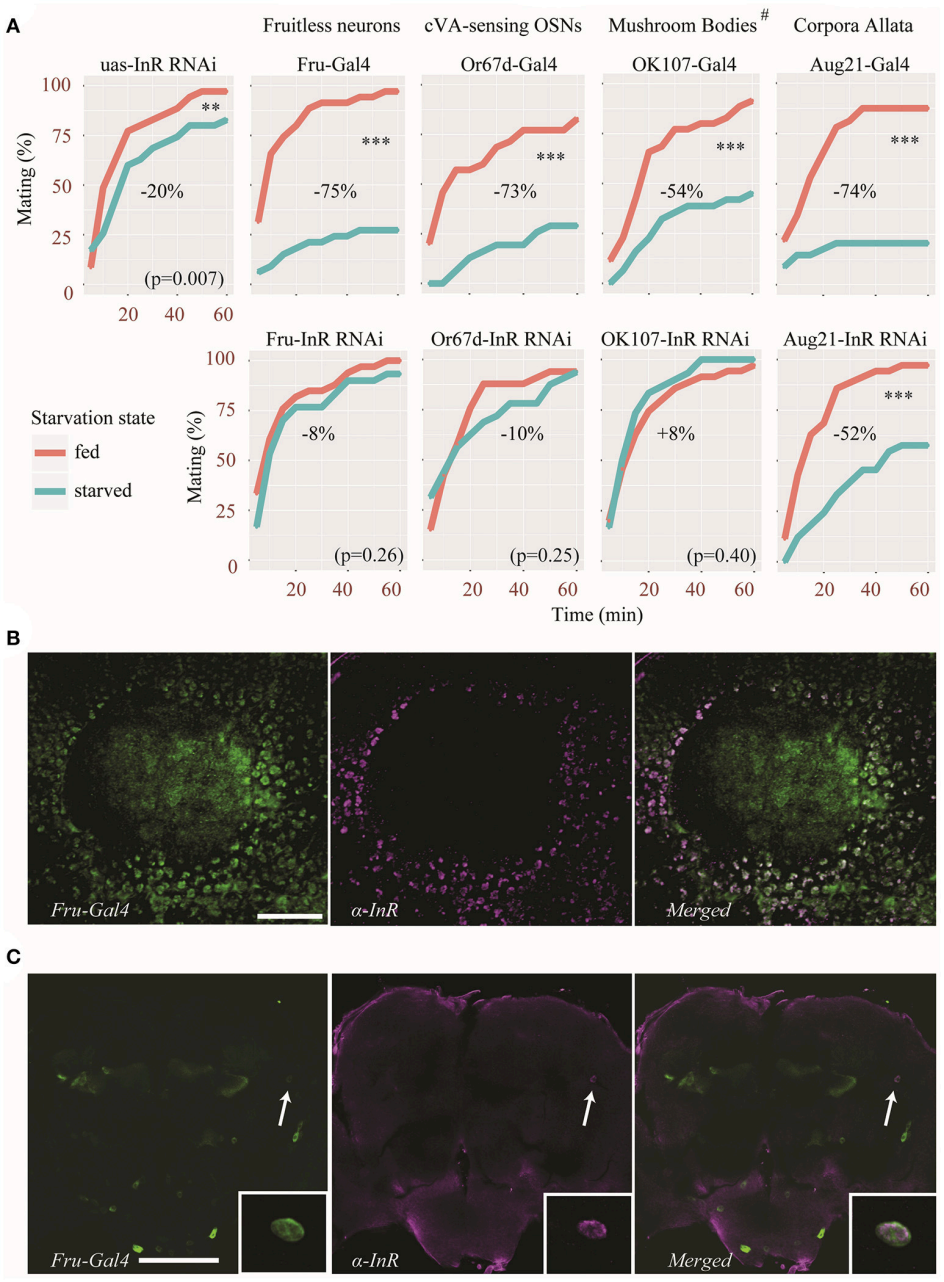
Insulin signaling acts on specific neuronal circuitry to regulate sexual receptivity during starvation. **(A)** An InR RNAi was expressed in Fruitless-positive neurons, the corpora allata, the mushroom bodies and cVA-sensing neurons, using specific Gal4 drivers. Top panels show the behavior of control parental lines. Bottom panels show the behavior of flies in which the InR RNAi was expressed. Estimated difference in sexual receptivity (based on the calculation of the area under curve) after starvation is indicated for each line. ^**^p < 0.01, ^***^p < 0.001 (Linear Mixed-Effect Model). ^#^OK107-Gal4 also drives expression to some extent to other parts of the brain (see text for details). **(B)** InR immunoreactivity (magenta) in Kenyon cells in the calyx (CA) of a mushroom body also showing *Fru*-Gal4 driven GFP expression (green). Scale bar = 20 μm. **(C)** Colocalization of InR immunoreactivity (magenta) in a pair of neurons showing *Fru*-Gal4 driven GFP expression (green) in the dorso-lateral protocerebrum (arrow). The inset shows a detail of the co-expression (from a different focal plane). Scale bar = 100 μm.

In all control lines (uas-InR RNAi and Gal4 lines) sexual receptivity was negatively affected by starvation (Figure 3A). However, the genetic background appears to have a substantial effect on the amplitude by which starvation affects female sexual receptivity (Figure 3A). Of note, the uas-InR RNAi line itself was only little affected (reduction of 20% of the sexual receptivity after starvation, Figure 3A). Nevertheless, knocking-down InR in Fruitless-positive neurons, or only in a subset of these neurons (Or67d-OSNs) reduced the effect of starvation on receptivity (with a reduction of only 8 to 10%). In fact, the difference in sexual receptivity between starved and fed females was not statistically significant in these flies. Similarly, when knocking-down InR in the mushroom bodies using the OK107-Gal4 driver, the effect of starvation was abolished. Interestingly, in this case, starved females were even slightly more receptive than fed females, although this effect was not statistically significant (increased receptivity of 8% in starved flies). In contrast, we did not observe any effect by expressing an InR RNAi in the corpora allata, with an effect intermediate to those of the two control lines (Figure 3A).

### InR is expressed in fruitless-positive neurons

Our behavioral analysis revealed that knocking down InR in *Fruitless*-positive neurons diminished the effect of starvation on female receptivity. We therefore performed immunostaining on fly brains in order to visualize which *Fruitless*-positive neurons express the insulin receptor. A large number of *Fruitless*-positive Kenyon cells were also immunoreactive to the insulin receptor antibody as can be observed in Figure 3B. In addition, we found InR immunolabeling in a pair of *Fruitless*-positive anterior dorso-lateral neurons (Figure 3C). Thus, at least in Kenyon cells of the mushroom bodies and in a few protocerebral cells, InR and *Fru* are co-expressed.

## Discussion

*Drosophila* females need nutrients to produce eggs and a nutrient rich substrate to lay their eggs (Drummond-Barbosa and Spradling, 2001; Becher et al., 2012). When food is scarce it would therefore be beneficial for flies to decrease their sexual behavior and to focus on food searching instead. On the other hand, female flies can store sperm and use it several days later when conditions are suitable (Qazi et al., 2003). It could therefore be optimal for females to remain receptive for short periods of food deprivation. Several insulin peptides produced in specific spatiotemporal patterns acting through one single receptor enables a fine-scale regulation of behaviors in response to changes in physiology. The expression of the different *dilps* is differentially affected by food quality or food deprivation (Bai et al., 2012; Whitaker et al., 2014; Post and Tatar, 2016). For instance, both starvation and dietary restriction reduce the expression of *dilp5* but increase the expression of *dilp6*, while the expression of *dilp2* is not affected by either condition (Bai et al., 2012; Whitaker et al., 2014). Our results suggest that DILP5 might be involved in the decrease of receptivity during non-feeding stages. Indeed, *dilp5* mutant females were less affected by starvation than other *dilp* mutants. The effect of the lack of DILP5 was no longer observed in the simultaneous absence of DILP2 and DILP3. Although, we cannot completely rule out background mutation effects, this suggests that DILP5 might interact with other DILPs to finely tune female sexual receptivity.

Insulin is known to act on the olfactory system to modulate odor sensitivity after feeding (Root et al., 2011). Moreover, normal InR expression in Or67d-expressing (Fruitless-positive) OSNs is necessary for fed females to be attracted to a blend of food odors and cVA (Lebreton et al., 2015), a pheromone promoting sexual receptivity (Kurtovic et al., 2007). Our results suggest that insulin signaling in Fruitless-positive neurons, and more specifically in Or67d OSNs may decrease sexual receptivity during starvation.

Fruitless-positive cells other than pheromone-sensing neurons can also be involved. We found different Fruitless-positive cells in the protocerebrum that strongly express InR. First of all, a large number of Kenyon cells in the calyx of the mushroom bodies express both Fruitless and the insulin receptor. Additionally, we found one pair of neurons with somata located in the anterior dorso-lateral protocerebrum. We could not trace any processes from these somata and do thus not know what neuropils they innervate. However, the fact that InR immunostaining was observed in Fruitless neurons, most of which were Kenyon cells, corroborate our behavioral results. Indeed, the sexual receptivity of females in which insulin signaling was knocked down in the mushroom bodies was not affected by starvation. Interestingly, the mushroom bodies are not required for virgin females to be receptive (Neckameyer, 1998), suggesting that these structures may regulate the activity of neuronal networks inducing sexual receptivity. However, this result must be take with caution, given the fact that the Gal4 line we used to target the mushroom bodies also drives expression to some extent in other brain tissues (Aso et al., 2009). Further experiments will be necessary to confirm that the mushroom bodies are indeed responsible for this effect.

Insulin signaling not only modulates neuronal activity in adults but also shapes neuronal networks during development (Song et al., 2003). The effects we observed in our study may therefore be the consequence of a developmental defect of specific neuronal circuitry rather than a direct effect of insulin on these neurons during starvation. However, Fruitless-positive neurons being required for females to be receptive (Kvitsiani and Dickson, 2006), we would expect fed females to be unreceptive if the disruption of insulin signaling had altered the connectivity of these neurons during development, which was not the case. This suggests that insulin acts on these neurons during adult stage to modulate sexual receptivity. This is different for the mushroom bodies, which are not necessary for females to be receptive (Neckameyer, 1998). Knocking down InR specifically during development or specifically in adults will be necessary to disentangle these two possible modes of action of insulin.

In contrast with Fruitless neurons and the mushroom bodies, we did not observe any effect of the corpora allata in the insulin-dependent control of sexual receptivity, whereas these structures have been linked to the development of receptivity in virgin females (Manning, 1966, 1967). This result should however be taken with caution, considering the behavioral variability displayed by the different transgenic lines, which would have prevented us from observing subtle changes. Nonetheless, our results suggest that the structures that generate behaviors (such as the corpora allata) and those modulating these behaviors (for example the mushroom bodies) can be different and the underlying mechanisms uncoupled.

Taken together, *Drosophila* flies adjust their sexual behavior to match their nutritional state. Together with other hormonal pathways (Lebreton et al., 2016), insulin regulates some aspects of sexual activity (Wigby et al., 2011; Kuo et al., 2012; Sakai et al., 2014; Watanabe and Sakai, 2015), both after food intake (Lebreton et al., 2015) and after a period of starvation. Our results suggest that specific insulin peptides regulate female receptivity, possibly by acting on pheromone perception at the periphery or directly in the central nervous system. Indeed, the mushroom bodies probably play a major role in the insulin-dependent effect of starvation on female sexual receptivity. The next step will be to untangle the specific neuronal circuitry involved.

## Ethics statement

All the experiments described in the manuscript were performed with laboratory-reared insects. No recommendations from an ethical committee were required.

## Author contributions

SL and PW conceived the project. SL designed, performed and analyzed the behavioral experiments. MC performed the immunostaining and the acquisition of confocal microscopy images. SL wrote the first draft of the manuscript. All authors contributed to the final version of the manuscript.

### Conflict of interest statement

The authors declare that the research was conducted in the absence of any commercial or financial relationships that could be construed as a potential conflict of interest. The reviewer ML and handling Editor declared their shared affiliation.
